# Supplementary figures and images for: A Novel Recessive Mutation in SPEG Causes Early Onset Dilated Cardiomyopathy
Source: PLoS Genet. 2020 Sep 14;16(9):e1009000. doi: 10.1371/journal.pgen.1009000 (PMC7571691; doi:10.1371/journal.pgen.1009000)

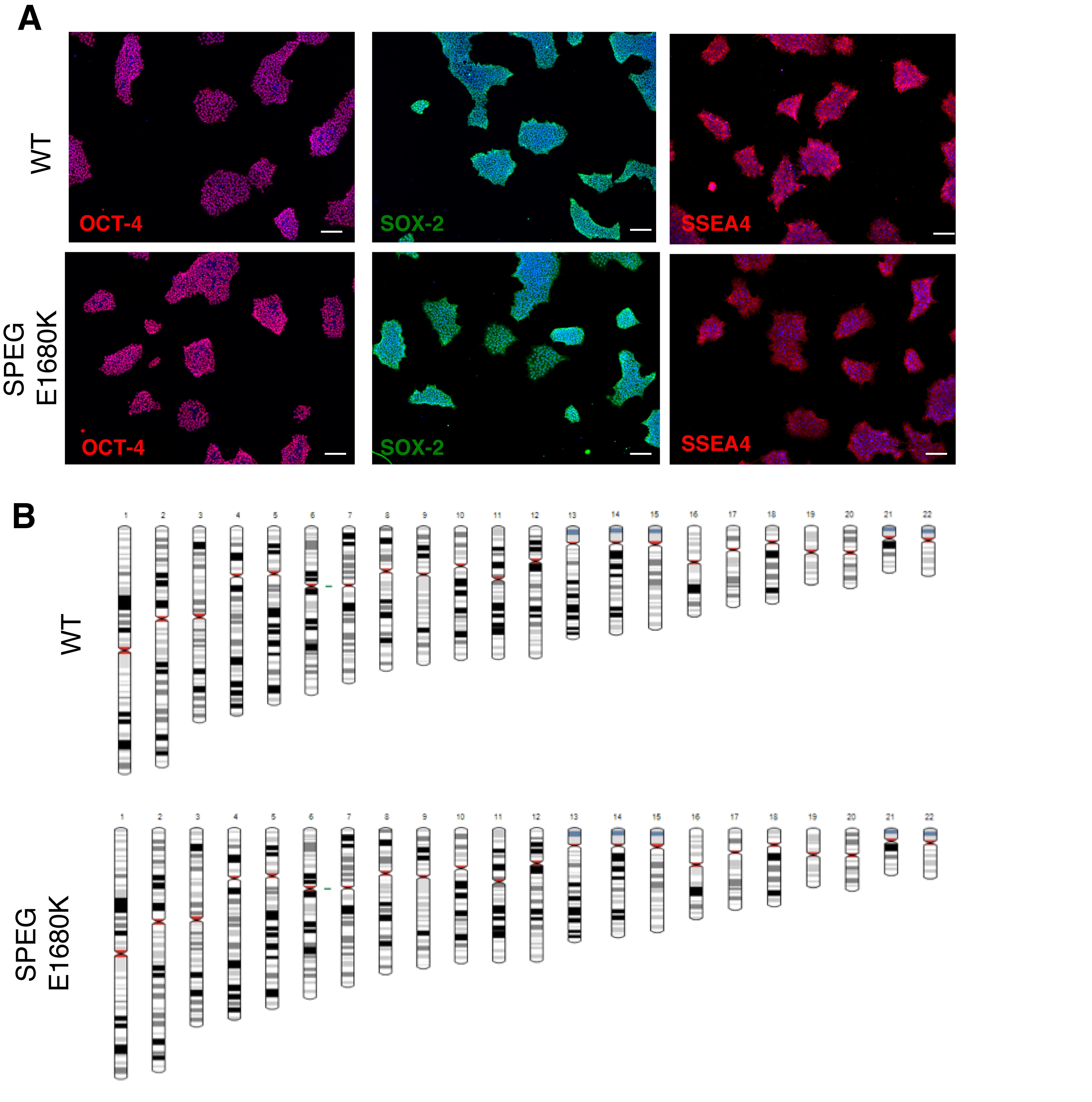

Supplement: S1 Fig — (A) Representative immunofluorescence images of patient-specific iPSC colonies immunostained for the pluripotency-associated markers OCT-4, SOX-2, and SSEA-4. Scale bar = 100μm. (B) SNP-based karyotype analysis of the isogenic iPSCs showing no karyotypic abnormalities. (TIF) [file pgen.1009000.s001.tif]

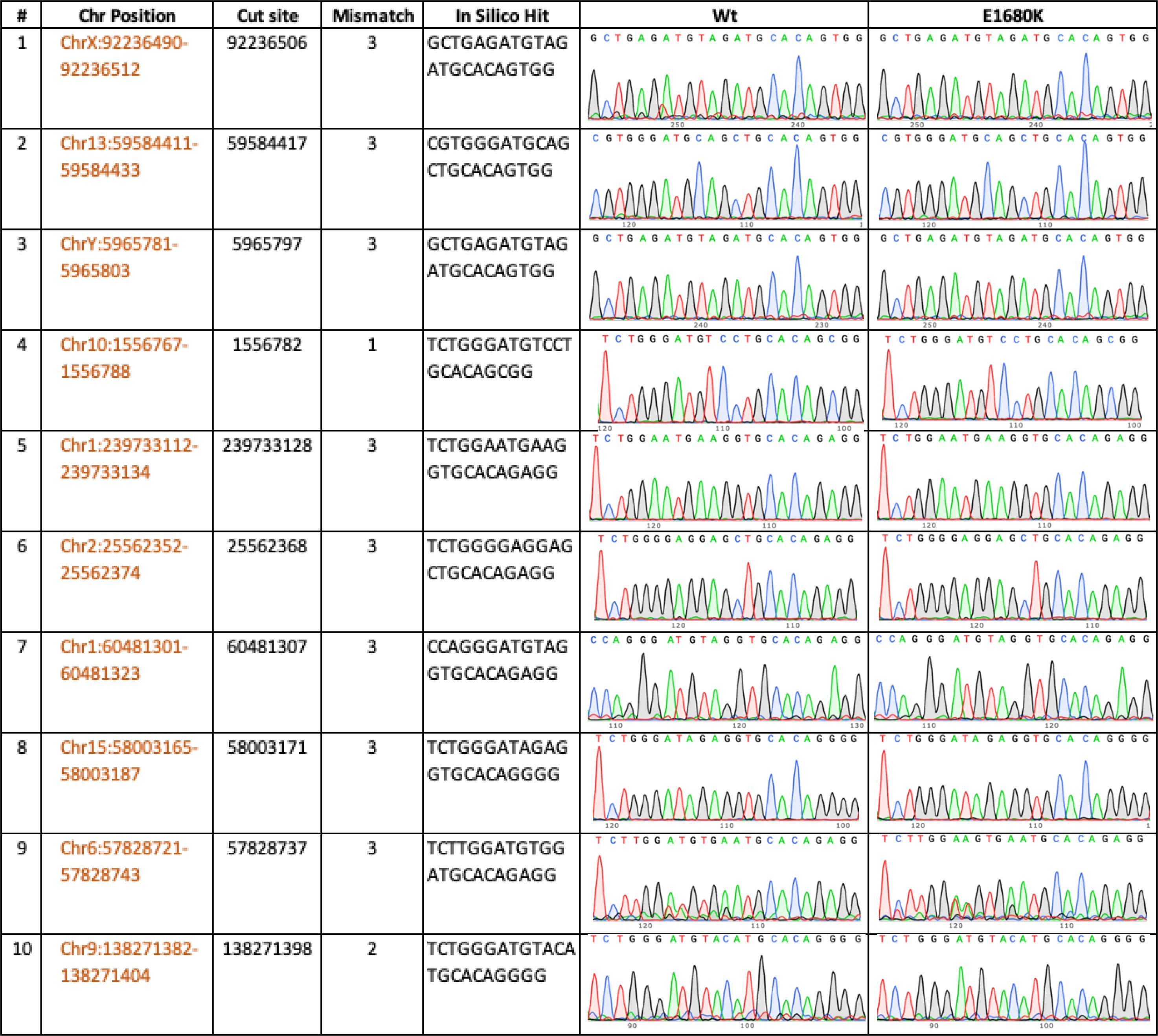

Supplement: S2 Fig — The top ten in silico predicted sites were amplified by PCR and analysed by Sanger sequencing. (TIF) [file pgen.1009000.s002.tif]

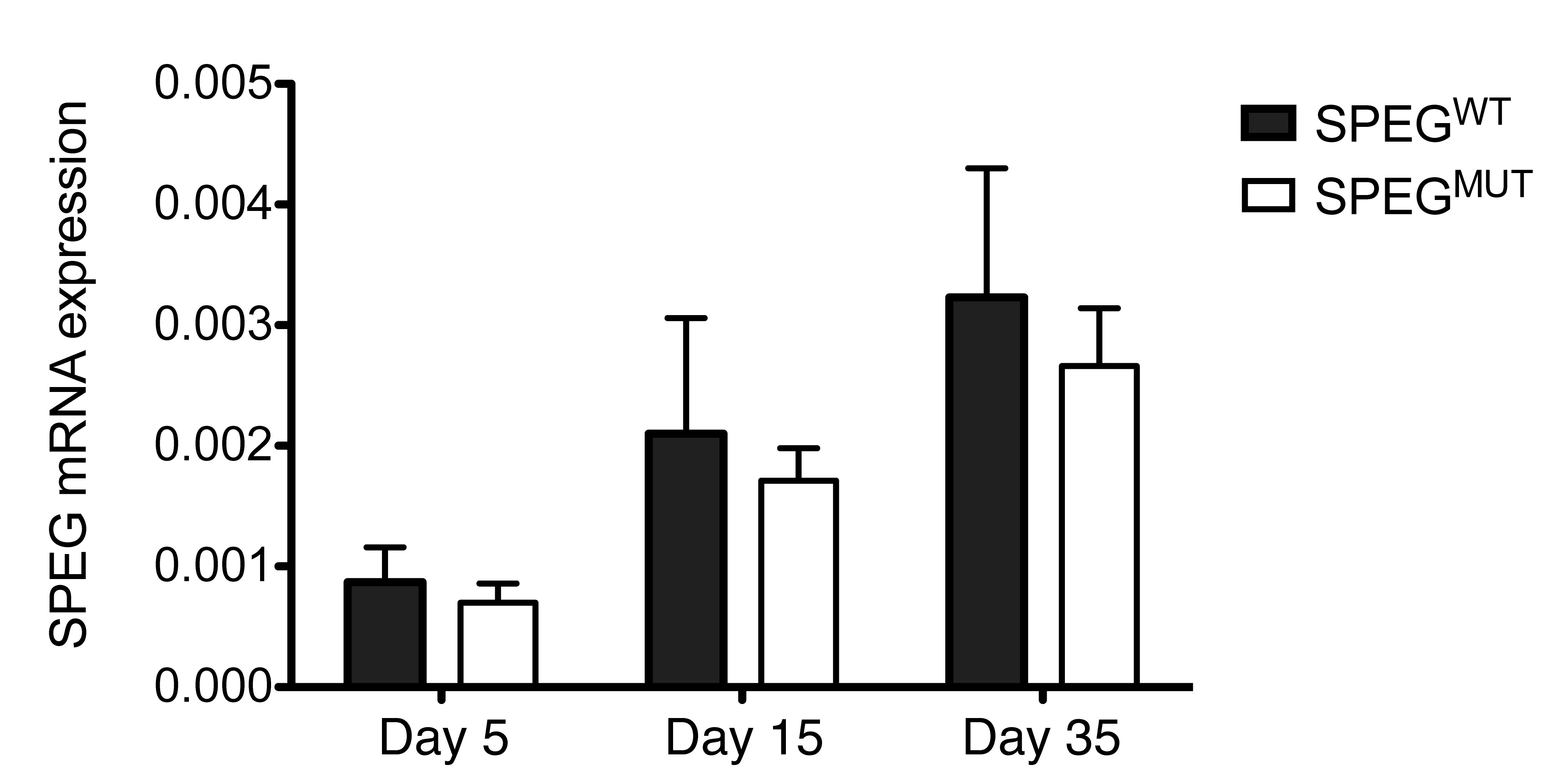

Supplement: S3 Fig — The expression levels of SPEG were measured by qPCR analyses at different stages of cardiac differentiation of wild type (WT) and E1680K mutant (MUT) as indicated. The expression levels are normalized to TNNT2 expression. Mean ± SD, n = 3 independent differentiation experiments. (TIFF) (TIF) [file pgen.1009000.s003.tif]

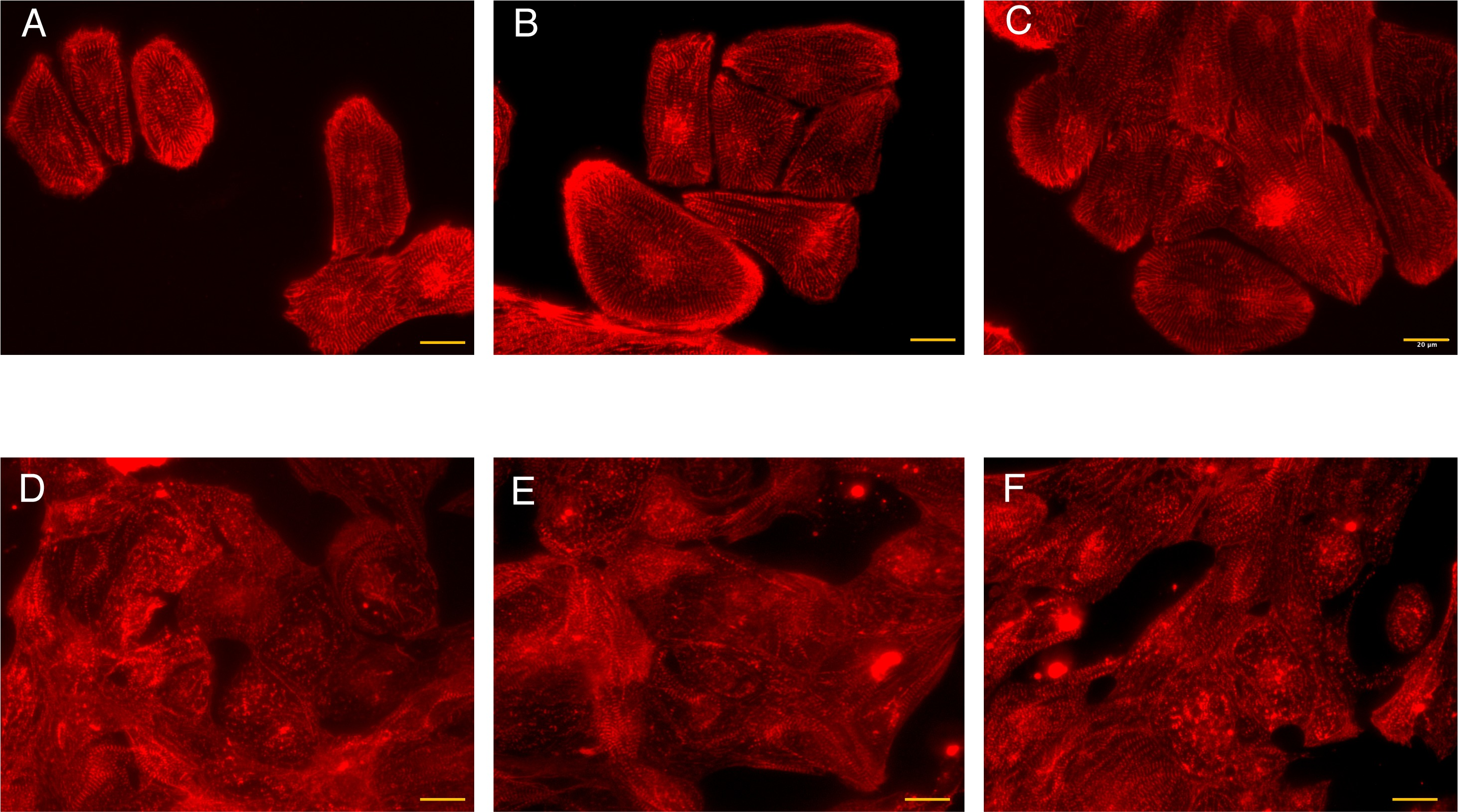

Supplement: S4 Fig — Representative immunofluorescent images of SPEGWT (A-C) and SPEGMUT (D-E) iPSC-CMs generated from 3 independent differentiation batches and used to quantify the sarcomere packing density associated with Fig 4D. (TIF) [file pgen.1009000.s004.tif]

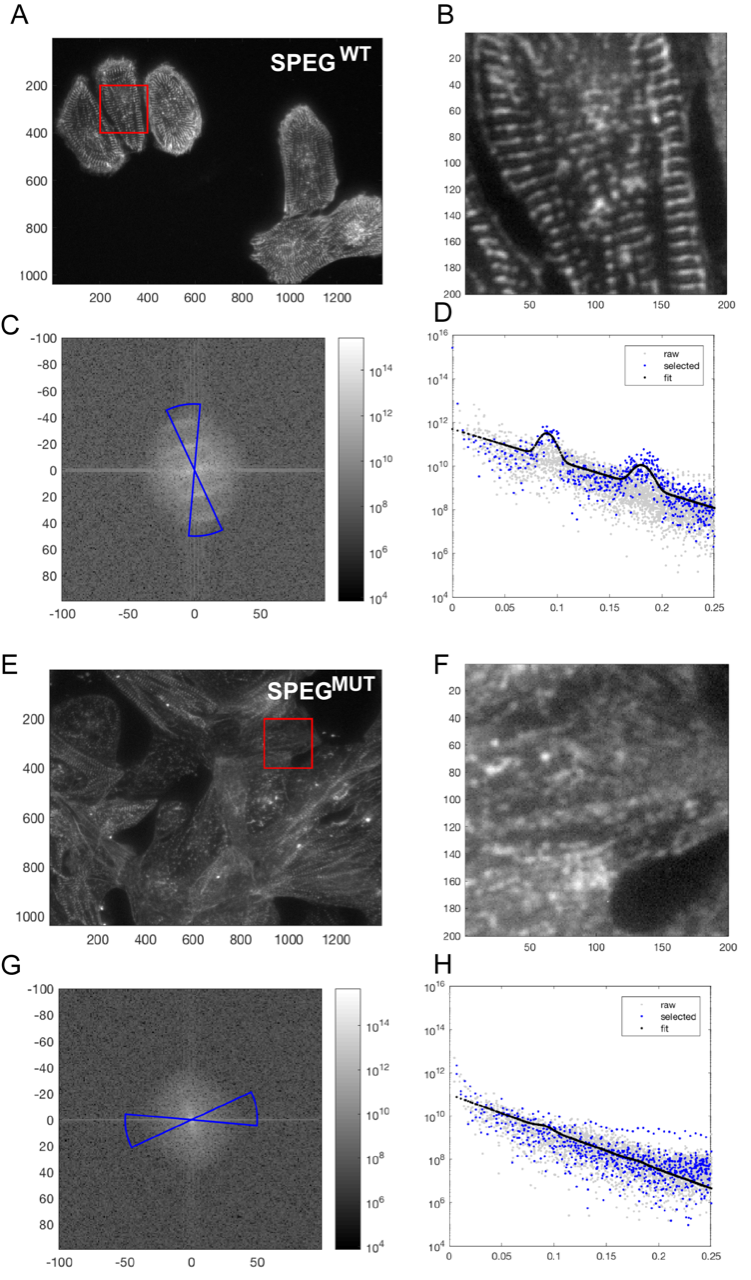

Supplement: S5 Fig — SPEGWT (panels A-D) and SPEGMUT (panels E-H). (A, E) Original image and selected region of interest (red square). (B, F) Enlarged image of the region of interest. (C, G) 2D Fourier power spectrum of the region of interest and selected orientation for 1D profile (blue wedges). (D, H) 1D profile of all data in the 2D spectrum (gray), selected orientation (blue), and fitted model (black). (TIF) [file pgen.1009000.s005.tif]
