# Supplementary material for: A Novel Recessive Mutation in SPEG Causes Early Onset Dilated Cardiomyopathy
Source: PLoS Genet. 2020 Sep 14;16(9):e1009000. doi: 10.1371/journal.pgen.1009000 (PMC7571691; doi:10.1371/journal.pgen.1009000)
Supplement: S1 Table — (DOCX) [file pgen.1009000.s006.docx]

**S1 Table. PCR Primers used for off target detection.**

| Site | PCR Primer Forward | PCR Primer Reverse |
| --- | --- | --- |
| 59584417 | AAATCCCTCTTCACTGCCCCAC | GAGCAGGTTTAGCAGACAGCTTTG |
| 25562368 | CTGCTTGGGCTGTGACAGGT | GGACCAGTCCACTCCTTGAGTCT |
| 60481307 | CAGAGTGAGACTCTGTCTGCC | CAGGCTGCCATAACAAAATACCGC |
| 92236506 | CTCCAGTTCCATCCATGTTGTTGC | GGGGAAACTAGAGACATGTGAACC |
| 5965797 | CTCCAGTTCCATCCATGTTGTTGC | GGGGAAACTAGAGACATGTGAACC |
| 239733128 | GGGAATGGGTAGCCTACTTGAG | GACTCAGATGGCTGCTAAGAAAACCAATCA |
| 58003171 | GAGGGTTCCGTGTGAGAGGT | GAAGGTGGTGGCACTATTAGGG |
| 57828737 | GAAGGGTGAGAGGCAAGATCAG | CCTCACTGTGACTGCCTTTTGC |
| 138271398 | GCCCCATGTGACTATTTGCGG | GCTGGTGGCATAAGCAACAGAAAC |
| 1556782 | CGGTCCAGTGCAGTAGCCAT | CGTCAGTTCTCCACTGCTTCTC |
