# Supplementary material for: A Novel Recessive Mutation in SPEG Causes Early Onset Dilated Cardiomyopathy
Source: PLoS Genet. 2020 Sep 14;16(9):e1009000. doi: 10.1371/journal.pgen.1009000 (PMC7571691; doi:10.1371/journal.pgen.1009000)
Supplement: S2 Table — (DOCX) [file pgen.1009000.s007.docx]

| Marker | PCR Primer Forward | PCR Primer Reverse |
| --- | --- | --- |
| D2S2944 | TCTGTCTTAGATGGATGAATGG | GGGATGGATGGAAAAGATTC |
| D2S2382 | ACATGCAACCGTGTGG | AGCACTCTTCAGTCCGAAC |
| D2S434 | TAAATCACTAGCCTTTGCCG | GCCATCTGTACTGTTCCCAG |
| rs3815849 | ACAACATCCCAGGAGACAGC | CCACCATTGACATGAACCTG |
| AC073838.6 | AGAGGCTCTCCCCCACAATA | CCTTTAGGAGGCTTGGGAAC |
| AC012510.8 | AGGGCATGGAGGAGAGAGAG | GCTGTATGCCTGATACCTTTCC |
| AC053503.1 | gcactggggtttgaagatg | tccatctctgtcctgcactg |
| AC053503.2 | CTCGCTAGGCTGCACCG | GCCTAACGAGCAGCCCC |
| D2S339 | GAAGTCATGGGAAGGC | AAGCAAAATGCAATCAGA |
| D2S102 | TAATCAAACCAAATGCTGGA | TCATCTTATACCATAAAGGC |
| AC017014.4 | TGCTGAGCCTGGTGATTACA | TGCCTAGGTAGAGGGGGAGT |
| AC019231.7 | TTGAGCATCCTTAAGAGACTTTGA | TTTGCCATGTATAATATGTTATTTTGA |

**S2 Table.** **Primers for PCR amplification of VNTRs and SNPs.**
