# Supplementary material for: A Novel Recessive Mutation in SPEG Causes Early Onset Dilated Cardiomyopathy
Source: PLoS Genet. 2020 Sep 14;16(9):e1009000. doi: 10.1371/journal.pgen.1009000 (PMC7571691; doi:10.1371/journal.pgen.1009000)
Supplement: Attachment — Submitted filename: plos genetics responses_final.docx [file pgen.1009000.s010.docx]

**Response to Reviewers’ critiques**

Thank you for the constructive critiques and acknowledging the challenges faced by researchers during these unprecedented times. Below are our point-by-point responses to reviewers’ comments. The corresponding changes in the manuscript text are highlighted in yellow.

**Editor**

*It is recognized in the present environment that the authors may not have access to the lab and therefore, may not be able to conduct the additional requested studies. Therefore, any revision would not require those experiments. However, that said the authors must make every effort to address all other concerns from the reviewers for the manuscript to be considered for publication. Explicitly, the authors must at the least:
1. Include all data not shown. This includes all data requested by the reviewers as well as all data referred to in the manuscript as not shown or not presented.*

**Response:** We made every effort to address the reviewers’ constructive criticism. In the revised manuscript we have provided all the available data requested by the reviewers and included a limitation section to outline the shortcomings of the current study.

*2. The authors must include all data with their isogenic lines. This is essential.*

**Response:**The data associated with the iPSC-CMs were generated from isogenic clones. We clarified this point in the revised manuscript per reviewer’s comment.

*3. The authors must include what data they have in hand to verify the cells are at the maturity they claim.*

**Response:** In the revised manuscript, we have clarified the maturation status of the cells and provided additional data in response to the reviewer’s comment.

4. *All text revisions by Reviewer #2.*

**Response:** All text revisions are addressed as detailed in our response to the reviewer

5. *All changes and edits and all response to reviewer’s must be accompanied by changes in the test of the manuscript and not solely in the letter that responds to the reviewer’s comments.*

**Response:** The responses to the reviewers’ comments were also address in the text of the manuscript as required

**Comments to the Authors:**

**Reviewer #1:**

*Dr. Levitas and colleagues propose that a novel mutation in the gene encoding striated muscle enriched protein kinase (SPEG) causes severe autosomal recessive dilated cardiomyopathy in a consanguineous family. They perform linkage analysis to identify SPEG then use CRISPR in human induced pluripotent stem cells to indicate that the SPEG E1680K mutation causes functional deficits consistent with the DCM phenotype. The study is novel, interesting and presented very clearly. A few concerns diminish enthusiasm for the manuscript in its present form.*

*1.The authors should cascade sequence SPEG in apparently unaffected parents and siblings to confirm the mode of inheritance and offer a sense of penetrance.*

**Response:** We thank the reviewer for this important comment. In the revised manuscript we clarified that we indeed sequenced all the parents and siblings for the SPEG mutation. All of the parents (II 1-6) are unaffected and heterozygotes for the SPEG c.5038G>A mutation, while the siblings that are affected are homozygous. The unaffected siblings are either heterozygous (III 2, 8 and 9) or carry a normal gene (III 7). The genotypes are provided in the **revised Figure 2**.

*2. Were the controls for the hIPSC-CM experiments actually isogenic controls as indicated? hIPSC-CMs with non-targeting gRNA in the vector construct would be more appropriate controls.*

**Response**: The genetically engineered hiPSCs differ exclusively at the edited locus and are otherwise isogenic. The ‘control’ iPSC-CMs were generated from an unedited clone derived from the same parental iPSC line as the SPEG mutant clone. The parental iPSC line was targeted with the CRISPR-Cas9 and gRNA vectors and we isolated an edited clone carrying a homozygous E1680K mutation (referred to as SPEG^MUT^) and another clone that was unmodified at the SPEG locus (referred to as SPEG^WT^). For clarity we updated the results section (**page 18**) and methods section (CRISPR/Cas9-mediated genome editing section, **page 7)** in the revised manuscript.

*3. Details regarding the maturity of the hIPSC-CMs is scant and the WT image in 5c does not look like a cardiomyocyte. How did the authors phenotype and validate their cells to assure maturity?*

The iPSC-CMs are relatively immature compared to adult cardiomyocytes and exhibit a fetal-like cardiomyocyte morphology [PMID: 26089365]. Despite this immaturity, iPSC-CMs is a suitable model for studying diseases that exhibit overt clinical phenotypes especially when the phenotype appears during fetal or early postnatal stages of development such as observed in the family in our study. This issue is further clarified in the revised manuscript (Introduction, **Page 4**)

Our standard ‘maturation’ protocol includes the extended culture of the differentiated cells (45-60 days) and expression analysis of cardiomyocyte-specific markers. In the revised manuscript we provided low magnification images displaying the typical cellular morphology of hiPSC-CMs. The cells were stained positive for alpha sarcomeric actinin, a cardiac-specific marker (Supplementary Figure 4). We also examined the contractile properties of in 3-dimensional (3D) engineered heart tissue (EHTs), a micro-engineered model with that exhibits a higher degree of maturation compared to 2D monolayer cultures, resembling human heart tissue [ PMID: 27211213] (**Figure 5**). All these details are clarified in the revised manuscript (Methods, **Page 6**; Results, **page 20**).

*4. The structural/histological E1680K phenotype in 5c is rather extreme—it is difficult to believe that these cells accurately represent the in vivo milieu. On a potentially related note, why did the authors choose to CRISPR the undifferentiated hiPSCs rather than hiPSC-CMs?*

In regard to the structural phenotype observed in our engineered model, sarcomere disarray is a common phenotype observed in several dilated cardiomyopathy iPSC models in vitro. [PMID: 26315439;  PMID: 31587567; PMID: 27105042; PMID: 26604136; PMID: 28941705]. However, there are limitations in using in vitro models and they not always recapitulate the in vivo mileu. Whether our observations represent the in vivo mileu in the SPEG mutant patients is not known. Evidence from histological examination of human hearts suggests that sarcomere disarray is associated with DCM (PMID: 1991369). In addition, studies in a mouse model suggest that loss of SPEG induces myofibril degeneration and sarcomere fragmentation leading to DCM [PMID: 19118250]. We can speculate that the SPEG mutation directly affects the sarcomere integrity and function, but we do not have any histological evidence. A new paragraph describing the limitations of the current study including the above considerations has been included in the revised manuscript (**Page 22**).

In regard to the second question, a key limitation of the CRISPR-cas9 genome editing is that human cells prefer the imprecise non-homologous end joining (NHEJ) pathway to repair a double strand break (DSB) rather than the more precise homologous DNA repair pathway using an exogenous repair template. Due to this pathway choice, editing events often result in NHEJ-mediated insertions and deletions at the DSB rather than the intended homology mediated modification. Thus, editing the hiPSC-CMs would result mainly in deletions and insertion events at the targeted locus rather than introducing the precise desired mutation. By targeting the hiPSCs, we can select isogenic pairs of disease-specific and control iPSC clones that differ exclusively at the disease-causing mutation. Parallel differentiation of these isogenic cell lines into cardiomyocytes provides the basis for the phenotypic analysis of disease-specific cellular pathologies. Phenotypes found in these cells can be directly attributed to the mutation and not the undesired NHEJ-mediated events. Detailed explanation of this issue can be found in several review articles and is beyond the scope of the present manuscript. However, we can add the above clarification to the manuscript, if the reviewer finds it critical.

*5. It would be relatively straightforward to immunblot for SERCA2a Thr484 phosphorylation to determine whether the E1680K mutation affects SERCA2a function, leading to the abnormalities in calcium handling.*

**Response:** We agree. Unfortunately, the antibody is not commercially available, and we cannot perform any experiments due to the laboratory shutdown. A new paragraph with the limitations of the current study, including the limited mechanistic insights, has been included in the revised manuscript (**Page 22**).

*6. Is the difference in diastolic calcium in Figure 5b actually statistically significant (p<0.001)?*

**Response:** There is a statistically significant difference between the mutant (n=120) and the isogenic control (n=130), p = 0.0062 (please see the statistical analysis in the table below). We have updated the **Figure 5** (panel b) to show the accurate p values of each panel and updated the figure legend accordingly (**P<0.01, ***P<0.001).

| Unpaired t test |  |
| --- | --- |
| P value | 0.0062 |
| P value summary | ** |
| Significantly different (P < 0.05)? | Yes |
| One- or two-tailed P value? | Two-tailed |
| t, df | t=2.763, df=248 |
|  |  |
| How big is the difference? |  |
| Mean of column A | 0.7667 |
| Mean of column D | 0.8074 |
| Difference between means (D - A) ± SEM | 0.04064 ± 0.01471 |
| 95% confidence interval | 0.01167 to 0.06960 |
| R squared (eta squared) | 0.02987 |

*7. Two of the affected children had associated congenital heart defects (VSD and ASD). Do the authors consider these defects to be associated with the SPEG mutation?*

Response: VSD and ASD are relatively common types of heart defects that present at birth (1 in 500 babies are born with VSD). In most children, the cause isn't known. It is unlikely that these findings, which were each seen in one individual patient, are associated with the SPEG mutation. However, we cannot totally rule out this possibility. A paragraph was added in the results section to clarify this issue (**Page 15**).

***Reviewer #2:*** *This manuscript provides strong clinical segregation data to support the contention that a recessive variant in SPEG, E1680K, is disease-causing for early onset DCM with childhood lethality in a consanguineous family. The methodologies for linkage analysis, homozygosity mapping and exome sequencing appear to be sound. The iPSC derivation and CM differentiation methodologies are also sound and the phenotype data support a pathogenic role of this recessive variant as there appears to be disruption of sarcomere structure and decreased contractility in unpatterned monolayers and in EHTs.

What is lacking from the manuscript is a mechanism for how this recessive variant reduces contractility and causes DCM. The authors speculate that it has something to do with phosphorylation of SERCA based on the increased Ca transients and faster Ca decay, but no data are provided to support this. Additional experimental data, if available, would add significant value. If these data are not available, and not attainable given current widespread laboratory shut downs, then a potential alternative is to add a limitation section that outlines the shortcomings of the current work and the limited functional characterization of these iPS-CM lines.*

**Response:** We agree that the major limitation of the current study is the lack of mechanistic link between the SPEG mutation and the pathogenesis of DCM. Unfortunately, we cannot perform any experiments due to the laboratory shutdowns. As suggested, a Limitation section is included at the end of the Discussion Section in the revised manuscript that outlines the shortcomings of the current study.

*1. The authors show that SPEG mRNA is not reduced in the iPSC-CMs with recessive E1680K variants. Do they have protein level data by western or mass spec? The variant could destabilize the protein even if the RNA is intact. This would be important to know whether this variant causes complete loss of functional protein vs some other effect on protein function*.

**Response:** We have tested three commercially available antibodies but were not able to detect the SPEG protein levels by western blot or immunofluorescence staining in iPSC-CMs. Mass spec is possible, but we do not have access to the laboratory and the core facility that could perform these experiments are currently closed.

*2. The claim that SPEG E1680K causes disruption of sarcomere architecture by IF is done in unpatterned cardiac myocytes which is not ideal, and further, not quantified in any objective manner. At the very least, multiple panels of different fields across at least 3 batches of differentiation with some form of semi-quantification should be shown*.

Response: We agree that the unpattern cells is not ideal. We are not able to provide pattern cell due to the laboratory shutdowns. In the revised manuscript, we provide additional images and quantified the degree of sarcomeric disarray using a custom MATLAB tool. All experiments reported in the study were done with three independent differentiations of the isogenic lines in parallel. For clarity we updated the figure legend accordingly to indicate that the data is represent n=3 independent differentiation batches. We added a note in the legend for clarity and provided a new supplementary figure showing the immunostaining (alpha-sarcomeric actinin) of three independent differentiation per line (**Supplementary Figure 4**). In addition, we provided quantification analysis of the sarcomeric architecture from all the images that we currently have available in the revised manuscript (**Figure 4d**). A new supplementary figure is also included that shows examples of the quantification analysis method (**Supplementary Figure 5**). We have updated the methods section in the revised manuscript (**Page 9**). The tool used for the analysis is also available to the scientific community (the MATLAB script is provided as a **Supplementary note**).

*3. The higher peak calcium transient and faster calcium decay kinetics are unusual for DCM. Typically in DCM, the SR load is reduced due to decreased activity/density of SERCA which leads to reduced transients. The authors do not measure SR load by caffeine application so it’s difficult to interpret these data. If they have additional calcium data, they should expand their results. If not, they need to speculate as to why these data differ from the vast majority of data on calcium dynamics in DCM and heart failure. Do they have any data on SERCA2a phosphorylation or PLN expression or phosphorylation?*

**Response:** We agree that end stage heart failure is associated with reduced Ca^2+^ transients and slower uptake kinetics. However, evidence in animal models of genetic DCM exhibit the opposite phenotype, similar to the observed phenotype in the SPEG mutant iPSC-CMs. For example, the cardiomyocytes of TnC I61Q TG and Tnnt2 K210del KI mice show increased peak amplitudes and faster relaxation kinetics. This phenotype is associated with a reduced calcium sensitivity of the myofilament, a hallmark of genetic DCM [PMID: 17556660; PMID: 27114035]. It is possible that the altered calcium dynamics observed in our SPEG model recapitulate, to some extent, the phenotypes observed in genetic models of DCM. We speculate that SPEG mutation could reduce calcium myofilament due to the sarcomere disarray or could affect PLN/Serca2a function and expression. In support of the latter, we have observed a significant decrease in the mRNA expression of PLN in the SPEG mutant compare to control iPSC-CMs, supporting the hypothesis that SPEG affects calcium dynamics by decreasing the PLN expression. New data on PLN expression is provided in **Figure 5b** and the discussion was revised accordingly in the revised manuscript (**Page 21**).

*4. A limitations section should be added that emphasizes the lack of mechanism based on the data presented, as well as the fact that homozygous variants are rare in DCM and that SPEG heterozygous variants may not necessarily be disease-causing. They should also soften the final summary paragraph that implies that DCM gene testing panels should be expanded based on this and other similar studies. The clinical segregation data for this example are very strong, but this level of clinical certainty is often not attainable. Functional data can be supportive but are not sufficient alone to establish pathogenicity of many variants identified in patients.*

**Response:** We thank the reviewer for these suggestions. A limitation section was included at the end of the Discussion Section in the revised manuscript that outlines the shortcomings of the current study. The final paragraph of the discussion was changed in the revised manuscript: “*This experimental framework combined with clinical segregation data could be broadly applied to distinguish genuine DCM disease-causing or disease-associated genetic variants from the broader genetic background where rare recessive variants are identified. A better understanding of the DCM etiology could ultimately improve our ability to care for patients at the bedside*.”

*5. Are there any objective clinical data for skeletal muscle function or injury in any of the patients? (i.e. like CKs?). If not, then they should soften the statement that the affected individuals had no clinical manifestations of myopathy.*

**Response:** The clinical evaluation of all the affected patient are consistent with normal skeletal muscle function. The patients have undergone extensive clinical testing and blood work over the years. The CK levels were normal. In **revised Table 1** we added the last CK value that are available for each patient in the record of the pediatric cardiology clinic. Patient #1, which died as a newborn did not have CK values in his records. However, all of the other patients had CK values within the normal range.

*6. Which parents do II-2 and 5 come from in the pedigree?*

**Response**: Thank you for pointing out the missing link in the pedigree. We have updated the family pedigree in the revised manuscript to indicate the parents of II-2 and 5 (**revised Figure 1 and Figure 2**).
